# EpCAM- and EGFR-Specific Antibody Drug Conjugates for Triple-Negative Breast Cancer Treatment

**DOI:** 10.3390/ijms23116122

**Published:** 2022-05-30

**Authors:** Chaoyu Zhang, Wenjie Sheng, Marwah Al-Rawe, T. M. Mohiuddin, Marcus Niebert, Felix Zeppernick, Ivo Meihold-Heerlein, Ahmad Fawzi Hussain

**Affiliations:** 1 Department of Gynecology and Obstetrics, Medical Faculty, Justus-Liebig-University Giessen, Klinikstr. 33, 35392 Giessen, Germany; chaoyu.zhang@med.uni-giessen.de (C.Z.); wenjie.sheng@med.uni-giessen.de (W.S.); marwah.al-rawe@gyn.med.uni-giessen.de (M.A.-R.); t.m.mohiuddin@gyn.med.uni-giessen.de (T.M.M.); felix.zeppernick@gyn.med.uni-giessen.de (F.Z.); ivo.meinhold-heerlein@gyn.med.uni-giessen.de (I.M.-H.); 2 Department of Molecular Cytology and Functional Genomics, Institute of Pathology, Justus-Liebig-University Giessen, Langhanssstr. 10, 35392 Giessen, Germany; marcus.niebert@patho.med.uni-giessen.de

**Keywords:** triple-negative breast cancer, tumor targeting therapy, antibody drug conjugate, SNAP-tag technology, EGFR, EpCAM

## Abstract

Triple-negative breast cancer (TNBC) is a group of heterogeneous and refractory breast cancers with the absence of estrogen receptor (ER), progesterone receptor (PgR) and epidermal growth factor receptor 2 (HER2). Over the past decade, antibody drug conjugates (ADCs) have ushered in a new era of targeting therapy. Since the epidermal growth factor receptor (EGFR) and epithelial cell adhesion molecule (EpCAM) are over expressed on triple-negative breast cancer, we developed novel ADCs by conjugating benzylguanine (BG)-modified monomethyl auristatin E (MMAE) to EpCAM- and EGFR-specific SNAP-tagged single chain antibody fragments (scFvs). Rapid and efficient conjugation was achieved by SNAP-tag technology. The binding and internalization properties of scFv-SNAP fusion proteins were confirmed by flow cytometry and fluorescence microscopy. The dose-dependent cytotoxicity was evaluated in cell lines expressing different levels of EGFR and EpCAM. Both ADCs showed specific cytotoxicity to EGFR or EpCAM positive cell lines via inducing apoptosis at a nanomolar concentration. Our study demonstrated that EGFR specific scFv-425-SNAP-BG-MMAE and EpCAM-specific scFv-EpCAM-SNAP-BG-MMAE could be promising ADCs for the treatment of TNBC.

## 1. Introduction

Female breast cancer is the leading cause of global cancer incidence worldwide in 2020 and ranks fifth in terms of cancer mortality [1]. Based on the expression of estrogen receptor (ER), progesterone receptor (PgR) and epidermal growth factor receptor 2 (HER2, also known as ERBB2), Perou and Sørlie’s team first classified breast cancer into five molecular subtypes: luminal subtype A; luminal subtype B; basal-like subtype; HER2 positive subtype; and normal breast-like subtype [2,3]. Triple negative breast cancer (TNBC) is characterized by the clinical absence of ER, PgR and HER2 diagnosed mainly via immunohistochemistry and accounts for 10–20% of all breast cancer [4]. Even though defined by different methods, TNBC is often, but not always, equal to the basal-like subtype, as most of the basal-like subtypes are TNBC and about 80% of TNBC are also basal-like breast cancer [5].

Patients positive for ER and/or PgR benefitted from endocrine therapy such as tamoxifen, which is confirmed to reduce recurrence rates throughout the first 10 years and mortality throughout the first 15 years [6]. Meanwhile, the application of HER2 targeted monoclonal antibodies (mAbs) such as pertuzumab or trastuzumab demonstrated a prolonged median overall survival (OS) of 16 months in patients with HER2-overexpressing breast cancer [7]. TNBC showed more aggressive biology compared with other subtypes with increased likelihood of distant recurrence and death ratio [8]. Due to the absence of hormone receptors and distinctive targetable antigens, TNBC is still struggling with limited therapeutic options including surgery and systematic chemotherapy [9].

One hundred years ago, Paul Ehrlich proposed a “magic bullet” concept aiming to target and eliminate tumor cells while sparing normal cells. Since then, numerous efforts had been made to find a tumor specific agent. From nitrogen mustard, to small molecules targeting mutated proteins, to multi-targeted inhibitors, antibodies now exhibit excellent potency for tumor-selective targeting [10]. Whereas most tumor-selective antibodies have limited therapeutic activity, antibody-drug conjugates (ADCs), which are made up of monoclonal antibodies tethered to cytotoxic agents, provide the possibility to achieve “magic bullet” activity by integrating the specific-binding ability of antibodies and tumor-killing capacity of cytotoxic agents.

The first generated ADCs rely on conjugating the cytotoxic agents to the antibodies using non-specific conjugation methods, which take advantage of naturally occurring amino acids such as lysine and cysteine without modification of antibodies, but result in an undesirable heterogeneous mixture of ADCs with varying drug to antibody ratios (DARs) [11]. In 2008, Junutula and co-workers produced homogeneous ADCs by engineering reactive cysteine residues site-specifically without disruption of interchain disulfide bonds, followed by cytotoxic drug attachment to these unpaired cysteines [12]. Since then, more and more site-specific conjugation methods have been developed, among which enzyme-based conjugation methods have emerged as promising alternative site-specific conjugation methods. SNAP-tag is a modified version of human DNA repair enzyme O6-alkylguanine-DNA-alkyltransferase (AGT), specifically reacting with O6-benzylguanine (BG) derivatives by irreversible transfer of an alkyl group to a cysteine residue and forms a stable thioether bond [13,14]. This site-specific conjugation strategy enabled the production of homogeneous ADCs with uniform pharmacokinetic properties.

Targeting therapy by means of ADCs emerged as a promising strategy to treat TNBC. Here, we developed a targeting method based on epidermal growth factor receptor (EGFR) and epithelial cell adhesion molecule (EpCAM), which have been reported to be overexpressed in TNBC patients [15,16,17]. In particular, EGFR associated with poor prognosis is more frequently overexpressed in TNBC than in other subtypes, ranging from 13% to 76% depending on population and methods of the evaluation and antibodies [15,18]. We genetically engineered fusion proteins consisting of SNAP-tag fused to single chain variable fragments (scFvs). The constructs are named scFv-425-SNAP (anti-EGFR) and scFv-EpCAM-SNAP (anti-EpCAM) respectively. Purified scFvs were then conjugated with BG-modified cytotoxic agent monomethyl auristatin E (MMAE) [19,20]. The specific cytotoxicity of both ADCs was confirmed in vitro. Compared to full-length antibodies, the small size (27 KDa) and short half-life (within hours) of scFvs improve the permeability of ADCs and reduce exposure time to normal cells which express low levels of targeting receptors. The scFvs-SNAP fusion proteins either labelled with fluorescence dyes or cytotoxic agents have already been evaluated in different tumors for imaging application or targeting therapy which exhibited impressive results, indicating the suitability of scFv-SNAP fusion proteins to be used as delivery vehicles [19,21,22].

Our study produced two MMAE-based ADCs with controlled stoichiometry targeting EGFR and EpCAM using SNAP-tag technology. The high-efficiency conjugation method, homogeneous products and selective cytotoxicity provide a promising novel targeting strategy for TNBC.

## 2. Results

### 2.1. Production of scFv-SNAP Fusion Proteins

The scFv-SNAP fusion proteins (~52 kDa) were expressed by HEK293T cells, and purified by an Ni-NTA superflow cartridge using the C-terminal 6 × His-tag. All fractions during purification were collected. After incubation with SNAP-Surface^®^ Alexa Fluor^®^ 488, the presence of fusion protein and the activity of SNAP-tag in collected fractions were confirmed in SDS-PAGE. ScFv-EpCAM-SNAP is shown as an example in Figure S1. ScFv-EpCAM-SNAP was present in supernatant. Proteins were eluted with 40 mM and 250 mM imidazole (Figure S1a). Considering that the amount of samples loaded in E2 were more than threefold that in E3, most proteins were eluted with 250 mM imidazole and proteins eluted with 40 mM imidazole were negligible. Coomassie brilliant blue stain indicated that only scFv-EpCAM-SNAP was eluted with 250 mM imidazole, and other proteins from the culture medium were removed stepwise (Figure S1b). ScFv-425-SNAP was produced and confirmed in the same way. This expression system and purification method yielded proteins up to 10 mg per liter of culture supernatant.

### 2.2. Conjugation of scFv-SNAP Fusion Proteins with BG-Modified Agents

Amino-PEG4-Val-Cit-PAB-MMAE (1370.7 Da) was modified with BG-GLA-NHS (481.5 Da) as described in methods. BG-GLA-PEG4-Val-Cit-PAB-MMAE (hereinafter called BG-MMAE) was purified and confirmed by high-performance liquid chromatography (HPLC) (Figure S2a–c) and mass spectrometry analysis (Figure S2d). The retention time for BG-GLA-NHS, Amino-PEG4-Val-Cit-PAB-MMAE and BG-NNAE was 1.607, 6.547 and 8.073 min, respectively. The mass spectrum analysis of BG-MMAE represented double and triple protonated product with total molecular weight 1852.787 Da, indicating the generation of a BG-modified cytotoxic agent, as expected.

**Figure 1:**
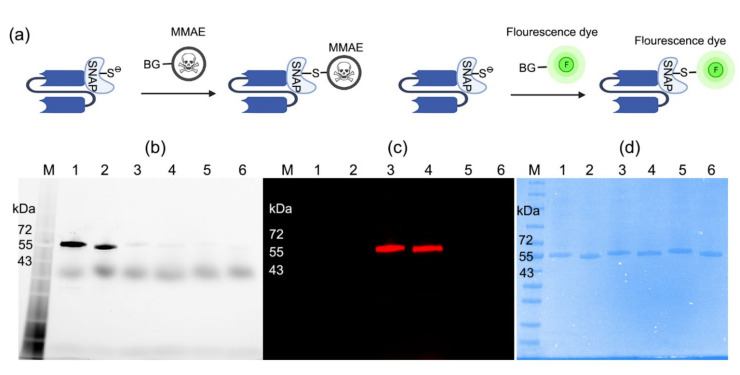
Conjugation of scFv-SNAP fusion proteins with BG-modified agents. (**a**) Schematic diagram of conjugation. ScFv-SNAP fusion proteins conjugated with SNAP-Surface^®^ Alexa Fluor^®^ 488 and visualized with ChemiDoc XRS+ System (**b**) or SNAP-Surface^®^ Alexa Fluor^®^ 647 visualized with Odyssey DLx Imager (**c**). (**d**) Corresponding Coomassie brilliant blue stain of SDS-PAGE. M: Blue prestained protein standard broad range (11−250 kDa). Lanes 1–2: scFv-425-SNAP and scFv-EpCAM-SNAP were incubated with SNAP-Surface^®^ Alexa Fluor^®^ 488. Lanes 3–4: the same proteins were labeled with SNAP-Surface^®^ Alexa Fluor^®^ 647, and were post-incubated with SNAP-Surface^®^ Alexa Fluor^®^ 488. Lanes 5–6: the same proteins were labeled with BG-MMAE, and were post-incubated with SNAP-Surface^®^ Alexa Fluor^®^ 488.

The conjugation of scFv-SNAP fusion proteins was further explored. ScFv-425-SNAP and scFv-EpCAM-SNAP were conjugated with BG-MMAE (used as therapeutic agents) or SNAP-Surface^®^ Alexa Fluor^®^ 647 (used as imaging agents) (Figure 1a). The site-specific conjugation was confirmed by post-incubation with SNAP-Surface^®^ Alexa Fluor^®^ 488. While unconjugated proteins retain the full activity to couple with SNAP-Surface^®^ Alexa Fluor^®^ 488, the specific coupling sites were blocked by SNAP-Surface^®^ Alexa Fluor^®^ 647, or BG-MMAE (Figure 1b). SNAP-Surface^®^ Alexa Fluor^®^ 488 was observed under UV light using ChemiDoc XRS+ System, and the SNAP-Surface^®^ Alexa Fluor^®^ 647 signal was observed at 685 nm using an Odyssey DLx Imager (Figure 1c). The presence of all proteins is shown in SDS-PAGE stained with Coomassie brilliant blue (Figure 1d). Our results demonstrated that BG-modified agents could be conjugated to scFv-SNAP fusion proteins site-specifically and sufficiently within 2 h at room temperature.

### 2.3. Binding and Internalization Properties of scFv-SNAP Fusion Proteins

**Figure 2:**
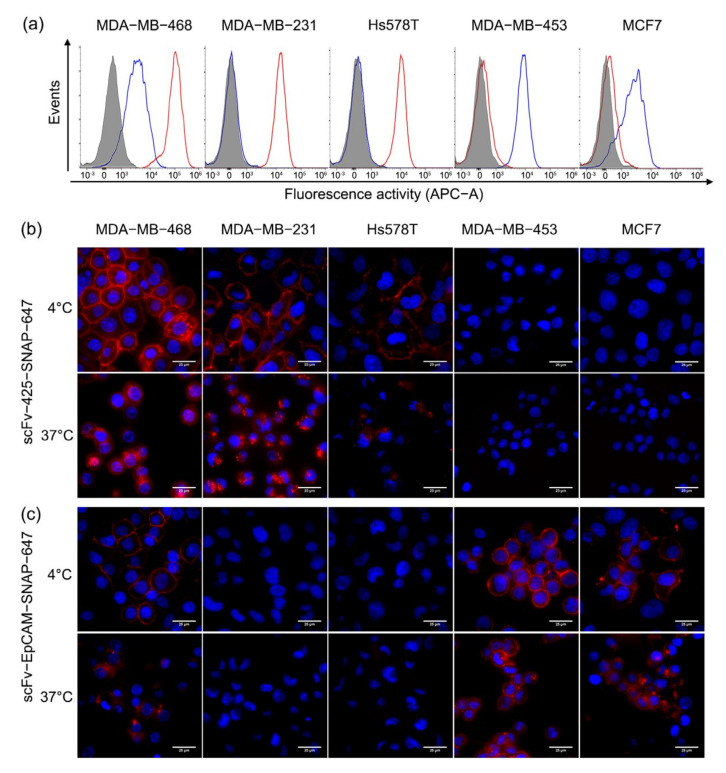
Specific binding and internalization of scFv-SNAP fusion proteins. (**a**) EGFR and EpCAM expression and specific targeting of Alexa Fluor^®^ 647-labeled scFv-SNAP fusion proteins were measured by flow cytometry. The red and blue curves represent cells treated with scFv-425-SNAP-647 or scfv-EpCAM-SNAP-647, respectively and the filled gray curve represents untreated cells. (**b**,**c**) The binding and internalization of scFv-425-SNAP-647 and scFv-EpCAM-SNAP-647 was visualized by fluorescence microscope. The binding process was carried out at 4 °C, while the internalization process was carried out at 37 °C. The red and blue signal represent Alexa Fluor^®^ 647-labeled scFv-SNAP fusion proteins and Hoechst 33,342 nuclear counterstain, respectively.

Targeting properties of Alexa Fluor^®^ 647-labeled scFv-425-SNAP and scFv-EpCAM-SNAP were validated by flow cytometry and fluorescence microscopy. Figure 2a shows that EGFR and EpCAM were differently expressed on four TNBC cell lines (MDA-MB-468, MDA-MB-231, MDA-MB-453 and Hs578T) and one ER positive cell line (MCF7), consistent with previous studies [23,24,25,26,27,28]. MDA-MB-468 expressed the highest level of EGFR, while MDA-MB-231 and Hs578T showed moderate expression of EGFR. MDA-MB-453 and MCF7 expressed minimal levels of EGFR. EpCAM was highly expressed on MDA-MB-453, MDA-MB-468 and MCF7, and minimally expressed on MDA-MB-231 and Hs578T. The binding was further confirmed by fluorescence microscopy at 4 °C (Figure 2b,c). ScFv-425-SNAP-647 (Figure 2b) and scFv-EpCAM-SNAP-647 (Figure 2c) bound to EGFR^high^ (MDA-MB-468, MDA-MB-231 and Hs578T) or EpCAM^high^ (MDA-MB-453, MCF7 and MDA-MB-468) cells lines. Meanwhile, unspecific binding was not observed on the EGFR^low^ (MDA-MB-453, MCF7) or EpCAM^low^ (MDA-MB-231 and Hs578T) cell lines.

Cells were incubated with Alexa Fluor^®^ 647-labeled proteins at 37 °C for 3 h to determine internalization (Figure 2b,c). The internalization of scFv-425-SNAP-647 was visibly observed on MDA-MB-468, MDA-MB-231 and Hs578T cell lines after 3 h, and was not observed in MDA-MB-453 and MCF7 as expected due to low expression of EGFR (Figure 2b). Likewise, scFv-EpCAM-SNAP-647 was internalized in MDA-MB-453, MCF7 and MDA-MB-468, expressing a high level of EpCAM over time, but showed slower internalization properties compared to scFv-425-SNAP-647 (Figure 2c).

### 2.4. Specific Cytotoxicity of MMAE Conjugated scFv-SNAP Fusion Proteins

**Figure 3:**
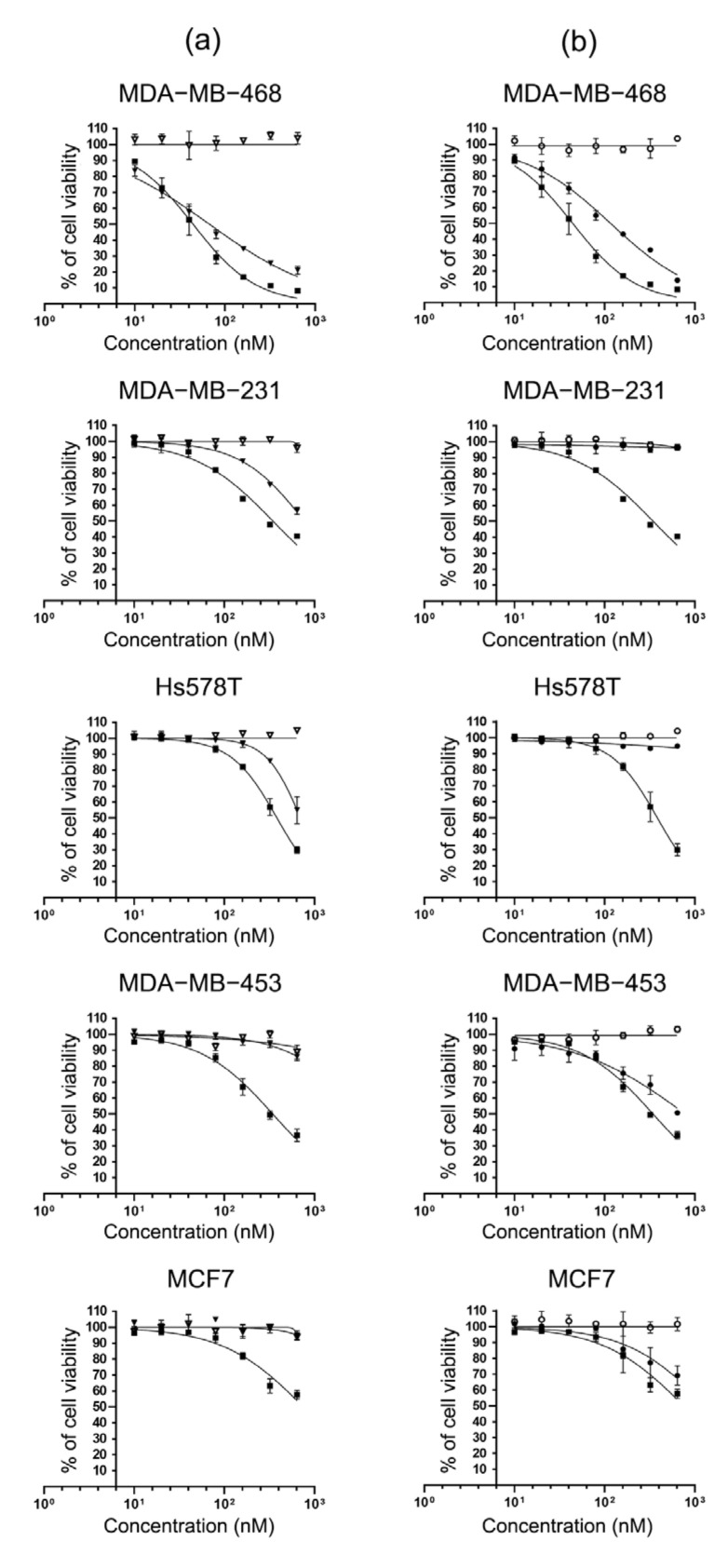
Specific cytotoxicity of MMAE conjugated scFv-SNAP fusion proteins. TNBC cell lines (MDA-MB-468, MDA-MB-231, Hs578T and MDA-MB-453) and ER^+^ cell line MCF7 were incubated with increasing concentrations (0, 10, 20, 40, 80, 160, 320, 640 nM) of MMAE, scFv-SNAP fusion proteins and corresponding ADCs at 37 °C for 72 h. (**a**) Cells treated with scFv-425-SNAP (▽), scFv-425-SNAP-MMAE (▼) and MMAE (■). (**b**) Cells treated with scFv-EpCAM-SNAP (⭘), scFv-EpCAM-SNAP-MMAE (●) and MMAE (■). Cytotoxicity was evaluated by cell viability assay. The experiment was carried out in triplicates at least three times. Data are shown as mean ± SD of one independent measurement.

Since the specific binding and internalization of scFv-SNAP fusion proteins have been confirmed, we expected that these properties were retained after conjugation with BG-MMAE and therefore a cytotoxicity assay was performed to determine the specific cytotoxicity of scFv-425-SNAP-MMAE and scFv-EpCAM-SNAP-MMAE. The five cell lines (MDA-MB-468, MDA-MB-231, MDA-MB-453, Hs758T and MCF-7) were incubated with increasing concentration of scFv-425-SNAP-MMAE, scFv-EpCAM-SNAP, scFv-425-SNAP, scFv-EpCAM-SNAP or MMAE. All TNBC cell lines were sensitive to MMAE, while MCF7 was relatively resistant. The specific cytotoxicity of scFv-425-SNAP-MMAE was observed in EGFR^high^ cell lines MDA-MB-468, MDA-MB-231 and Hs578T, but not in EGFR^low^ cell lines MDA-MB-453 and MCF7 (Figure 3a). Likewise, the specific cytotoxicity of scFv-EpCAM-SNAP-MMAE was confirmed in EpCAM^high^ cell lines MDA-MB-453, MDA-MB-468 and MCF7, but not in EpCAM^low^ cell lines MDA-MB-231 and Hs578T (Figure 3b). Meanwhile, the unconjugated scFv-SNAP fusion protein did not affect viability in any cell line. The IC_50_ values of scFv-425-SNAP-MMAE were in the range of 114.7–740.4 nM, while scFv-EpCAM-SNAP-MMAE were in the range of 135.2–981.7 nM. Full details are listed in Table 1.

### 2.5. Induction of Apoptosis by MMAE Conjugated scFv-SNAP Fusion Proteins

**Figure 4:**
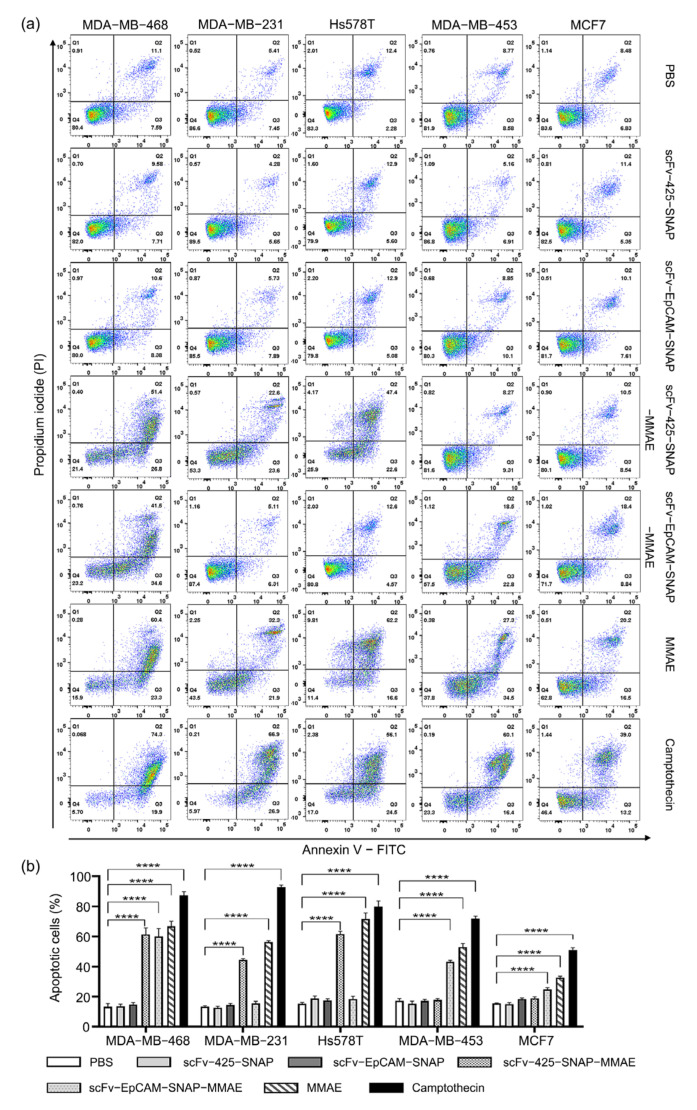
Induction of apoptosis by MMAE, scFv-SNAP fusion proteins and corresponding ADCs. (**a**) Scatter plot of one measurement as an example. (**b**) The sum of early and late apoptotic cells from three independent experiments carried out in triplicate. Data are shown as mean ± SEM. The statistical analysis was carried out using one-way ANOVA and corrected by Tukey’s test (**** *p* ≤ 0.001).

We further explored whether scFv-425-SNAP-MMAE and scFv-EpCAM-SNAP-MMAE triggered apoptosis using an Annexin V/PI assay (Figure 4). Cells were incubated with 640 nM of MMAE, scFv-425-SNAP-MMAE, scFv-EpCAM-SNAP-MMAE, scFv-425-SNAP or scFv-EpCAM-SNAP for 48 h. Cells treated with PBS were used as negative control, and camptothecin which was a commonly used cytotoxic agent to trigger apoptosis was used as a positive control. MMAE induced apoptosis in all cell lines compared to negative control. After incubation with scFv-425-SNAP-MMAE, the proportion of apoptotic cells were significantly increased in the EGFR^high^ cell lines MDA-MB-468, MDA-MB-231 and Hs578T, but not in the EGFR^low^ cell lines MDA-MB-453 and MCF7. Similarly, scFv-EpCAM-SNAP-MMAE induced apoptosis in target cell lines MDA-MB-453, MDA-MB-468 and MCF7, but not in the EpCAM^low^ cell lines MDA-MB-231 and Hs578T. Unconjugated scFv-SNAP fusion proteins did not induce apoptosis in any tested cell line.

## 3. Discussion

TNBC is a heterogeneous, aggressive type of breast cancer associated with limited treatment options and comparably poor prognosis. Although immunotherapy in combination with chemotherapy showed promising activity in PD-L1-expressing TNBC, systematic chemotherapy still remains the standard for TNBC treatment [9]. The emergence and development of targeted therapy has dramatically changed the management of antitumor therapy. Monoclonal antibodies and small molecule drugs are the most common types of targeted therapy. Albeit a superior selective binding property, only a few mAbs exhibit moderate antitumor activity by themselves and are often used in combination with other anticancer drugs. Meanwhile, most small molecular weight cytotoxins, which are commonly used in chemotherapy, have potent cytotoxicity, but suffer from rapid plasma clearance and low specificity. ADCs assuredly widened the scope of mAb-based targeting therapy by endowing mAbs with cytotoxicity and retaining high specificity. To date, there are approximately one hundred mAb products and twelve ADCs have been approved by the FDA (U.S. Food and Drug Administration), among which three mAbs and three ADCs are designed to treat breast cancer [29,30]. Of note, only sacituzumab and govitecan are targeting the human trophoblast cell-surface antigen 2 (Trop-2) in metastatic TNBC, while all others are targeting Her2. Since sacituzumab govitecan was the first and sole ADC for TNBC, our study estimated the specific cytotoxicity of two other ADCs for TNBC.

An ideal ADC consists of three core components: a highly specific mAb targeting tumor-associated antigen expressed on the tumor surface with minimal expression on non-malignant cells, a stable and flexible linker that can survive during blood circulation and release cytotoxic agents effectively at target sites, and a potent cytotoxic payload. The majority of ADCs are built on full-length antibodies, especially IgG1, limiting tumor penetration due to their large molecular size of approximately 150 kDa [31,32]. The half-life for IgG1, IgG2 and IgG4 is around 21 days, and the mAbs were found to be retained in circulation instead of targeted tumor sites [32]. The long half-life and poor permeability of these drug conjugated mAbs increase the exposure risk of normal cells to toxic drugs and cause nonspecific cell death during circulation [33]. Compared to full-length antibodies, scFvs consist exclusively of variable fragments of heavy and light chain, resulting in a much smaller molecular weight (27 kDa). The smaller size extends the possibility of antibodies to penetrate tissues, while variable regions are still capable of binding to antigens. Nevertheless, given that antibodies less than 25 kDa can be fast filtered by glomerulus, scFv exhibit blood half-life for only 11 min and whole body half-life for 1.4 h [34,35]. Fusing of the scFv to SNAP-tag improves its half-life by increasing the size of the antibody to around 52 kDa, and ∼50 kDa has been proven to be the optimal size to obtain a maximum tumor-to-plasma exposure ratio [36]. Furthermore, ADCs are supposed to be internalized rapidly to avoid off-target effects. The internalization time varies from antibody to antibody. Our flow cytometry and fluorescence microscopy have proven that both conjugated scFv-425-SNAP and scFv-EpCAM-SNAP could specifically bind to EGFR or EpCAM overexpressing cell lines, respectively, and be rapidly internalized within 3 h, indicating the usability of these scFvs as delivery vehicles.

The mechanisms of therapeutic mAbs action are mainly due to signal transduction changes, antibody-dependent cellular cytotoxicity (ADCC), complement-dependent cytotoxicity (CDC) and antibody-dependent cellular phagocytosis (ADCP) [37,38,39]. The immune reactions triggered by therapeutic mAbs are highly associated with Fc domains. However, the immune responses might be superfluous for mAbs in ADCs and even lead to adverse effects in an FcR-dependent manner [40,41]. The efficacy of ADCs is mainly conducted by highly potent cytotoxic drugs such as auristatin, calicheamicin, maytansinoid, camptothecin and pyrrolobenzodiazepine dimer. Auristatins are commonly used payloads, accounting for five out of twelve of the current ADCs approved by the FDA and six out of fourteen approved worldwide [29]. Mechanistically, it destabilizes microtubules and blocks the microtubule assembly by binding tubulin, causing G2/M phase cell cycle arrest in the nanomolar concentration range [42]. We demonstrated the potent cytotoxicity of MMAE in four TNBC cell lines (MDA-MB-468, MDA-MB-231, Hs578T and MDA-MB-453) and an ER^+^ cell line MCF7, followed with the modification of MMAE with benzylguanine and then conjugated to scFv-SNAP fusion proteins. The cytotoxicity of scFv-SNAP-MMAE was dependent on the drug susceptibility and antigen expression level of cell lines. According to our cytotoxicity assays of four TNBC cell lines (Figure 3), MDA-MB-468 (IC_50_: 61.16 nM) displayed a high sensitivity to MMAE, while MDA-MB-231, Hs578T and MDA-MB-453 (IC_50_: 344.3 nM, 314.7 nM and 311.5 nM, respectively) were moderately sensitive. Consistent with the EGFR and EpCAM expression level confirmed by flow cytometry (Figure 2a), scFv-SNAP-MMAE exhibited comparable cytotoxicity with the IC_50_ values in the range of 114.7–740.4 nM in EGFR^high^ and EpCAM^high^ TNBC cell lines. Most importantly, unspecific cytotoxicity was not observed in the EGFR^low^ and EpCAM^low^ cell lines. Considering the high drug susceptibility and high expression of EGFR and EpCAM, MDA-MB-468 showed the most outstanding response to scFv-425-SNAP-MMAE (IC_50_: 114.7 nM) and scFv-EpCAM-SNAP-MMAE (IC_50_: 135.2 nM). Of note, MCF7 was not as sensitive as other cell lines to MMAE, with an IC_50_ value of 660.6 nM, but it still manifested specific cytotoxity to scFv-EpCAM-SNAP-MMAE (IC_50_: 981.7 nM). Even though MCF7 is not a TNBC cell line, TNBC is a heterogeneous cancer and drug resistance should be taken into consideration. Conjugating different payloads such as DNA cleavage agents, microtubule inhibitors and TOPO1 inhibitors might be a potential way to solve the drug resistance.

Linkers that connect the cytotoxin to the mAb maintain the stability of ADCs in systemic circulation and play a key role in pharmacokinetic and therapeutic properties [43,44]. Generally, linkers can be divided into cleavable and non-cleavable linkers. Cleavable linkers increase the possibility of a “bystander” effect, which means the released cytotoxins kill surrounding cells expressing low or no ADC-targeted antigens. Dipeptide-containing linker valine-citrulline (val-cit) used in our experiment is the most popular enzymatic cleavable linker used in current clinical research, and Brentuximab vedotin typically used for lymphoma treatment is a case in point [11,45].

In addition to three core components mentioned above, the conjugation method is also crucial for ADCs. The conventional drug conjugation methods are either using lysine sidechains or cysteine residues on the mAb backbone, yielding over 10^6^ different ADC species with different DARs and pharmacological properties [43,46]. Site-specific conjugation methods enable the generation of homogeneous products with defined DAR and unified pharmacokinetics, as exemplified by non-natural amino acids incorporation, engineered cysteine conjugation, and enzymatic conjugation [47]. Nevertheless, some site-specific conjugation methods require complicated procedures such as redox reaction and metal-dependent catalytic reaction, or huge amounts of cytotoxic agents [48,49,50]. These may affect the activity of antibodies and are also cost-prohibitive. The SNAP-tag technology we used here is an enzymatic conjugation method. Apart from site-specific conjugation and product homogeneity, this self-labeling tag achieved rapid and efficient reaction under physiological conditions with a 1:1 stoichiometry [13]. Theoretically, ADCs with more drug molecules are supposed to achieve higher cytotoxicity. However, Hamblett et al. revealed that mAb with reduced drug loading could increase the therapeutic index [51]. The research group compared the antitumor activity of mAb with two, four or eight MMAE molecules (E2, E4, and E8, respectively) in vitro and in vivo. The drug potency was undoubtedly improved with increased drug loading when applied to cell lines. However, a xenograft model revealed unexpected results when E4 showed comparable effectiveness with E8 at same mAb doses, and E2 exhibited the best response after doubling the dose to reach an equal amount of MMAE with E4 and E8 [51]. In this study we conjugated scFv-SNAP fusion proteins with one MMAE molecule and confirmed that conjugation could be fulfilled with a two-fold molar excess of BG derivatives within 2 h at room temperature. The SNAP-tag technology along with scalable recombinant proteins production could facilitate an economic production of ADCs. Overall, our research established two homogeneous ADCs based on SNAP-tag technology, targeting the overexpressed antigens EGFR and EpCAM in TNBC. Compared to current available ADCs, highly potent cytotoxic drugs armed to scFv are more likely to be delivered into deep tumor regions due to their smaller size, and their short half-life improves the safety profile in the meantime. Combined with SNAP-tag technology, both of the scFv-SNAP-MMAE exhibited high specificity, rapid internalization and specific cytotoxicity in TNBC cell lines, indicating the promising application of scFv and SNAP-tag in the ADC field. Despite the impressive results, our study still needs to be further confirmed in vivo, and pharmacokinetic properties should be determined. Additionally, given the drug susceptibility as we mentioned above, SNAP-tag could also facilitate the coupling of different or more toxic agents to overcome drug resistance or improve cytotoxicity.

## 4. Materials and Methods

### 4.1. Cell Culture

All medium formulations were supplemented with 10% (v/v) fetal bovine serum and 100 U/mL penicillin-streptomycin. The TNBC cell lines MDA-MB-468 (ACC 738), MDA-MB-231 (ACC 732), MDA-MB-453 (ACC 65), Hs578T (ACC 781) and ER^+^ human breast cancer cell line MCF-7 (ACC 115) were cultured in DMEM medium. The human embryonic kidney cell line HEK293T was cultured in RPMI 1640 (Gibco). Cells were incubated at 37 °C in a humidified atmosphere containing 5% CO_2_. All cell lines were purchased from the Leibniz Institute DSMZ-German Collection of Microorganisms and Cell Cultures (Braunschweig, Germany). Medium and additives were purchased from Invitrogen, Darmstadt, Germany.

### 4.2. SNAP-Fusion Protein Expression and Purification

The scFv-SNAP fusion proteins were expressed as previously described [13]. Briefly, the SNAP-tag and scFv fragment were inserted into the mammalian expression vector pMS. The plasmids were transfected into HEK293T cells using Roti^®^ Fect (Carl Roth, Karlsruhe, Germany). Transfected cells were selected by zeocin (InvivoGen, Toulouse, France) (0.1 mg/mL) supplemented in medium. ScFv-425-SNAP and scFv-EpCAM-SNAP were secreted into culture medium, and then isolated from the supernatant with different imidazole concentrations (10, 40 and 250 mM, respectively) using an Ni-NTA Superflow cartridge (Qiagen, Hilden, Germany) on an ÄKTA start system (GE Healthcare Bio-Sciences AB, Uppsala, Sweden,). All eluted fractions were collected during purification. After incubation with SNAP-Surface^®^ Alexa Fluor^®^ 488 (New Englands Biolabs, Ipswich, MA, USA) at room temperature for 20 min in the dark, fractions were run in 10% SDS-PAGE to confirm the activity of SNAP-tag and the presence of proteins followed by Coomassie brilliant blue staining.

### 4.3. Synthesis of Benzylguanine-Modified Cytotoxic Drug

Amino-PEG4-Val-Cit-PAB-MMAE (BroadPharm, San Diego, CA, USA) and BG-GLA-NHS (New England Biolabs, Ipswich, MA, USA) were dissolved in DMSO. Amino-PEG4-Val-Cit-PAB-MMAE was incubated with a two-fold molar excess of BG-GLA-NHS in 1 × phosphate buffered saline (PBS) at room temperature for 4 h. BG-modified MMAE was analyzed and purified by HPLC via a Eurospher II 100-5 C18 column (8 × 250 mm, 5 µm, 100 Å) (Knauer, Berlin, Germany) at a flow rate of 4 mL/min. Separations were carried out using 40% acetonitrile, 60% water, and 0.1M ammonium acetate (4 mL/min/isocratic). The mass of BG-GLA-PEG4-Val-Cit-PAB-MMAE was confirmed using a Bruker MicroTOF LC mass spectrometer with an electrospray ion source. Accurate masses were derived from mass spectra in the range 0–2000 m/z.

### 4.4. Conjugation of scFv-SNAP Fusion Proteins with Benzylguanine-Modified Agents

BG-modified or SNAP-Surface^®^ Alexa Fluor^®^ 647 (New Englands Biolabs, Ipswich, MA, USA) were conjugated to scFv-SANP fusion proteins by incubating at a 2:1 molar ratio for 2 h at room temperature in the dark. The residual agents were removed by 40K MWCO Zeba ™ Spin Desalting Columns (Thermo Fisher Scientific, Rockford, IL, USA). The site-specific conjugation was confirmed by post-incubation with SNAP-Surface^®^ Alexa Fluor^®^ 488. SNAP-Surface^®^ Alexa Fluor^®^ 488 and SNAP-Surface^®^ Alexa Fluor^®^ 647 fluorescent were visualized under UV light using ChemiDoc XRS+ System (BIO-RAD, Hercules, CA, USA) and Odyssey DLx Imager (LI-COR Biosciences, Bad Homburg, Germany) individually after protein separation in SDS-PAGE.

### 4.5. Flow Cytometry

ScFv-SNAP fusion protein was conjugated with SNAP-Surface^®^ Alexa Fluor^®^ 647 instead of a cytotoxic agent to visualize cell-binding activity. The uman breast cancer cell lines MDA-MB-468, MDA-MB-231, MDA-MB-453, Hs578T and MCF-7 were used in flow cytometry. Here, 4 × 10^5^ cells were washed with 1 mL PBS twice, and incubated with 1 µg of each SNAP-Surface^®^ Alexa Fluor^®^ 647 labeled SNAP-tag fusion protein in 200 µL of PBS for 30 min on ice. After two washing steps, cells were resuspended in 200 µL of PBS and analyzed by CytoFLEX Flow Cytometers (Beckman Coulter, Indianapolis, IN, USA). Data was analyzed in FlowJo 10.7.1 (Becton, Dickinson & Company, Ashland, OR, USA).

### 4.6. Fluorescence Microscopy

MDA-MB-468, MDA-MB-231, MDA-MB-453, Hs578T and MCF-7 were seeded in black 96-well plate with a clear bottom (Greiner Bio-One, Frickenhausen, Germany) to a density of 40,000 cells/well and incubated overnight at 37 °C. To validate the internalization, cells were washed with PBS two times, and then incubated with 1 µg of each SNAP-Surface^®^ Alexa Fluor^®^ 647 labeled scFv-SNAP fusion protein at 37 °C for 3 h. After two washing steps, cells were incubated with Hoechst 33,342 fluorescent nuclear counterstain (1:500 in PBS) (Thermo Fisher Scientific, Darmstadt, Germany) for 15 min at 37 °C. Cells were washed with PBS twice and incubated in 100 µL of PBS. To determine the binding property, cells were fixed before incubation with antibodies and all steps were carried out at 4 °C. Briefly, cells were fixed by 4% formaldehyde solution for 10 min, followed by two washing steps with PBS. Residual formaldehyde was quenched by incubating with 50 nM ammonium chloride for 5 min. The internalization and binding were visualized with a DMi8 S Live-cell microscope (Leica Microsystems, Wetzlar, Germany) using a 100× oil objective.

### 4.7. Cytotoxic Assay

Cytotoxicity of MMAE based ADCs was evaluated using a Cell Proliferation (XTT) Kit II (Roche, Mannheim, Germany). MDA-MB-468, MDA-MB-231, MDA-MB-453, Hs758T and MCF-7 were seeded in 96-well plates at a density of 5000 cells/well in 50 µL of culture medium and incubated at 37 °C overnight, followed by incubation with serially diluted scFv-425-SNAP, scFv-EpCAM-SNAP, scFv-425-SNAP-MMAE, scFv-EpCAM-SNAP-MMAE or MMAE (0, 10, 20, 40, 80, 160, 320, 640 nM) at 37 °C for 72 h. Cells incubated with 25% DMSO were set as toxic control. Viability was determined by incubating cells with a 50 µL XTT labeling mixture at 37 °C for 4 h. The substrate conversion was monitored at a 450 nm absorbance wavelength and 650 nm reference wavelength using an Infinite^®^ M Plex microplate reader (Tecan, Grödig, Austria). The experiment was repeated independently three times in triplicates.

### 4.8. Induction of Apoptosis

The induction of apoptosis was determined by Alexa Fluor^®^ 488 annexin V/dead cell apoptosis kit (Thermo Fisher Scientific, Eugene, OR, USA) using the manufacturer’s instructions. Briefly, MDA-MB-468, MDA-MB-231, MDA-MB-453, Hs758T and MCF-7 were seeded in 24-well plates at a density of 50,000 cells/well in triplicates, and then incubated with 640 nM of scFv-425-SNAP, scFv-EpCAM-SNAP, scFv-425-SNAP-MMAE, scFv-EpCAM-SNAP-MMAE or MMAE at 37 °C for 48 h. Cells treated with PBS or camptothecin (Merck KGaA, Darmstadt, Germany) were used as negative or positive control, respectively. Cells were washed by cold PBS after harvesting, and then resuspended in 100 µL of 1 X annexin-binding buffer (10 mM HEPES, 140 nM NaCl, 2.5 mM CaCl2, pH 7.4). After incubation with Alexa Fluor^®^ 488 annexin V and propidium iodide (PI), early and late apoptotic cells were detected on BD FACSCanto ^TM^ II Flow Cytometer(BD Biosciences, San Jose, CA, USA). The experiment was repeated independently three times in triplicate.

### 4.9. Statistical Analysis

All experiments were carried out independently for at least three times in triplicate. The half maximal inhibitory concentration (IC_50_) was calculated by GraphPad Prism 9.0.0 (GraphPad Software, San Diego, CA, USA) and shown as mean ± SEM. The data of induction of apoptosis was presented as mean ± SEM and significance was calculated by one-way analysis of variance (ANOVA) followed by Tukey’s test using GraphPad Prism 9.0.0 (**** *p* ≤ 0.0001).

## Figures and Tables

**Table 1:** IC_50_ values (nM) of MMAE, scFv-SNAP fusion proteins and corresponding ADCs.

Cell Lines	MMAE	scFv-425-SNAP-MMAE	scFv-EpCAM-SNAP-MMAE	scFv-425-SNAP	scFv-EpCAM-SNAP
MDA-MB-468	61.16 ± 2.433	114.7 ± 7.14	135.2 ± 9.6	-	-
MDA-MB-231	344.3 ± 13.46	738.3 ± 11.2	-	-	-
Hs578T	314.7 ± 12.35	740.4 ± 17.55	-	-	-
MDA-MB-453	311.5 ± 15.47	-	551 ± 30.76	-	-
MCF7	660.6 ± 35.22	-	981.7 ± 35.02	-	-

The IC_50_ values indicate the concentration that inhibit cell viability by 50% relative to untreated control cells. The data represented three independent experiments carried out in triplicate and were presented as mean ± standard error of the mean (SEM).

## Data Availability

Not applicable.

## Supplementary Materials

The following supporting information can be downloaded at: www.mdpi.com/article/10.3390/ijms23116122/s1.

## References

[1] Sung H., Ferlay J., Siegel R.L., Laversanne M., Soerjomataram I., Jemal A., Bray F. (2021). Global Cancer Statistics 2020: GLOBOCAN Estimates of Incidence and Mortality Worldwide for 36 Cancers in 185 Countries. CA Cancer J. Clin..

[2] Perou C.M., Sorlie T., Eisen M.B., van de Rijn M., Jeffrey S.S., Rees C.A., Pollack J.R., Ross D.T., Johnsen H., Akslen L.A. (2000). Molecular portraits of human breast tumours. Nature.

[3] Sorlie T., Perou C.M., Tibshirani R., Aas T., Geisler S., Johnsen H., Hastie T., Eisen M.B., van de Rijn M., Jeffrey S.S. (2001). Gene expression patterns of breast carcinomas distinguish tumor subclasses with clinical implications. Proc. Natl. Acad. Sci. USA.

[4] Garrido-Castro A.C., Lin N.U., Polyak K. (2019). Insights into Molecular Classifications of Triple-Negative Breast Cancer: Improving Patient Selection for Treatment. Cancer Discov..

[5] Foulkes W.D., Smith I.E., Reis-Filho J.S. (2010). Triple-negative breast cancer. N. Engl. J. Med..

[6] Davies C., Godwin J., Gray R., Clarke M., Cutter D., Darby S., McGale P., Pan H.C., Taylor C., Early Breast Cancer Trialists’ Collaborative Group (2011). Relevance of breast cancer hormone receptors and other factors to the efficacy of adjuvant tamoxifen: Patient-level meta-analysis of randomised trials. Lancet.

[7] Kunte S., Abraham J., Montero A.J. (2020). Novel HER2-targeted therapies for HER2-positive metastatic breast cancer. Cancer.

[8] Dent R., Trudeau M., Pritchard K.I., Hanna W.M., Kahn H.K., Sawka C.A., Lickley L.A., Rawlinson E., Sun P., Narod S.A. (2007). Triple-negative breast cancer: Clinical features and patterns of recurrence. Clin. Cancer Res..

[9] Gradishar W.J., Anderson B.O., Abraham J., Aft R., Agnese D., Allison K.H., Blair S.L., Burstein H.J., Dang C., Elias A.D. (2020). Breast Cancer, Version 3.2020, NCCN Clinical Practice Guidelines in Oncology. J. Natl. Compr. Cancer Netw..

[10] Strebhardt K., Ullrich A. (2008). Paul Ehrlich’s magic bullet concept: 100 years of progress. Nat. Rev. Cancer.

[11] McCombs J.R., Owen S.C. (2015). Antibody drug conjugates: Design and selection of linker, payload and conjugation chemistry. AAPS J..

[12] Junutula J.R., Raab H., Clark S., Bhakta S., Leipold D.D., Weir S., Chen Y., Simpson M., Tsai S.P., Dennis M.S. (2008). Site-specific conjugation of a cytotoxic drug to an antibody improves the therapeutic index. Nat. Biotechnol..

[13] Hussain A.F., Heppenstall P.A., Kampmeier F., Meinhold-Heerlein I., Barth S. (2019). One-step site-specific antibody fragment auto-conjugation using SNAP-tag technology. Nat. Protoc..

[14] Kolberg K., Puettmann C., Pardo A., Fitting J., Barth S. (2013). SNAP-tag technology: A general introduction. Curr. Pharm. Des..

[15] Nakai K., Hung M.C., Yamaguchi H. (2016). A perspective on anti-EGFR therapies targeting triple-negative breast cancer. Am. J. Cancer Res..

[16] Abd El-Maqsoud N.M., Abd El-Rehim D.M. (2014). Clinicopathologic implications of EpCAM and Sox2 expression in breast cancer. Clin. Breast Cancer.

[17] Soysal S.D., Muenst S., Barbie T., Fleming T., Gao F., Spizzo G., Oertli D., Viehl C.T., Obermann E.C., Gillanders W.E. (2013). EpCAM expression varies significantly and is differentially associated with prognosis in the luminal B HER2(+), basal-like, and HER2 intrinsic subtypes of breast cancer. Br. J. Cancer.

[18] Masuda H., Zhang D., Bartholomeusz C., Doihara H., Hortobagyi G.N., Ueno N.T. (2012). Role of epidermal growth factor receptor in breast cancer. Breast Cancer Res. Treat..

[19] Kampmeier F., Niesen J., Koers A., Ribbert M., Brecht A., Fischer R., Kiessling F., Barth S., Thepen T. (2010). Rapid optical imaging of EGF receptor expression with a single-chain antibody SNAP-tag fusion protein. Eur. J. Nucl. Med. Mol. Imaging.

[20] Amoury M., Kolberg K., Pham A.T., Hristodorov D., Mladenov R., Di Fiore S., Helfrich W., Kiessling F., Fischer R., Pardo A. (2016). Granzyme B-based cytolytic fusion protein targeting EpCAM specifically kills triple negative breast cancer cells in vitro and inhibits tumor growth in a subcutaneous mouse tumor model. Cancer Lett..

[21] Woitok M., Klose D., Di Fiore S., Richter W., Stein C., Gresch G., Grieger E., Barth S., Fischer R., Kolberg K. (2017). Comparison of a mouse and a novel human scFv-SNAP-auristatin F drug conjugate with potent activity against EGFR-overexpressing human solid tumor cells. Onco Targets Ther..

[22] Kessler C., Pardo A., Tur M.K., Gattenlohner S., Fischer R., Kolberg K., Barth S. (2017). Novel PSCA targeting scFv-fusion proteins for diagnosis and immunotherapy of prostate cancer. J. Cancer Res. Clin. Oncol..

[23] Martowicz A., Spizzo G., Gastl G., Untergasser G. (2012). Phenotype-dependent effects of EpCAM expression on growth and invasion of human breast cancer cell lines. BMC Cancer.

[24] Mostert B., Kraan J., Bolt-de Vries J., van der Spoel P., Sieuwerts A.M., Schutte M., Timmermans A.M., Foekens R., Martens J.W., Gratama J.W. (2011). Detection of circulating tumor cells in breast cancer may improve through enrichment with anti-CD146. Breast Cancer Res. Treat..

[25] Wang G., Benasutti H., Jones J.F., Shi G., Benchimol M., Pingle S., Kesari S., Yeh Y., Hsieh L.E., Liu Y.T. (2018). Isolation of Breast cancer CTCs with multitargeted buoyant immunomicrobubbles. Colloids Surf. B Biointerfaces.

[26] Bhattacharyya S., Kurdziel K., Wei L., Riffle L., Kaur G., Hill G.C., Jacobs P.M., Tatum J.L., Doroshow J.H., Kalen J.D. (2013). Zirconium-89 labeled panitumumab: A potential immuno-PET probe for HER1-expressing carcinomas. Nucl. Med. Biol..

[27] Stanley A., Ashrafi G.H., Seddon A.M., Modjtahedi H. (2017). Synergistic effects of various Her inhibitors in combination with IGF-1R, C-MET and Src targeting agents in breast cancer cell lines. Sci. Rep..

[28] Yi Y.W., You K., Bae E.J., Kwak S.J., Seong Y.S., Bae I. (2015). Dual inhibition of EGFR and MET induces synthetic lethality in triple-negative breast cancer cells through downregulation of ribosomal protein S6. Int. J. Oncol..

[29] Fu Z., Li S., Han S., Shi C., Zhang Y. (2022). Antibody drug conjugate: The “biological missile” for targeted cancer therapy. Signal Transduct. Target. Ther..

[30] Mullard A. (2021). FDA approves 100th monoclonal antibody product. Nat. Rev. Drug Discov..

[31] Shin T.H., Sung E.S., Kim Y.J., Kim K.S., Kim S.H., Kim S.K., Lee Y.D., Kim Y.S. (2014). Enhancement of the tumor penetration of monoclonal antibody by fusion of a neuropilin-targeting peptide improves the antitumor efficacy. Mol. Cancer Ther..

[32] Drago J.Z., Modi S., Chandarlapaty S. (2021). Unlocking the potential of antibody-drug conjugates for cancer therapy. Nat. Rev. Clin. Oncol..

[33] Turner C.T., McInnes S.J.P., Voelcker N.H., Cowin A.J. (2015). Therapeutic Potential of Inorganic Nanoparticles for the Delivery of Monoclonal Antibodies. J. Nanomater..

[34] Flynn A.A., Pedley R.B., Green A.J., Dearling J.L., El-Emir E., Boxer G.M., Boden R., Begent R.H. (2003). The nonuniformity of antibody distribution in the kidney and its influence on dosimetry. Radiat. Res..

[35] Choi H.S., Liu W., Misra P., Tanaka E., Zimmer J.P., Itty Ipe B., Bawendi M.G., Frangioni J.V. (2007). Renal clearance of quantum dots. Nat. Biotechnol..

[36] Li Z., Li Y., Chang H.P., Chang H.Y., Guo L., Shah D.K. (2019). Effect of Size on Solid Tumor Disposition of Protein Therapeutics. Drug Metab. Dispos..

[37] Adams G.P., Weiner L.M. (2005). Monoclonal antibody therapy of cancer. Nat. Biotechnol..

[38] Scott A.M., Wolchok J.D., Old L.J. (2012). Antibody therapy of cancer. Nat. Rev. Cancer.

[39] Shah A., Rauth S., Aithal A., Kaur S., Ganguly K., Orzechowski C., Varshney G.C., Jain M., Batra S.K. (2021). The Current Landscape of Antibody-based Therapies in Solid Malignancies. Theranostics.

[40] Tolcher A.W. (2020). The Evolution of Antibody-Drug Conjugates: A Positive Inflexion Point. Am. Soc. Clin. Oncol. Educ. Book.

[41] Uppal H., Doudement E., Mahapatra K., Darbonne W.C., Bumbaca D., Shen B.Q., Du X., Saad O., Bowles K., Olsen S. (2015). Potential mechanisms for thrombocytopenia development with trastuzumab emtansine (T-DM1). Clin. Cancer Res..

[42] Sapra P., Shor B. (2013). Monoclonal antibody-based therapies in cancer: Advances and challenges. Pharmacol. Ther..

[43] Diamantis N., Banerji U. (2016). Antibody-drug conjugates--an emerging class of cancer treatment. Br. J. Cancer.

[44] Chau C.H., Steeg P.S., Figg W.D. (2019). Antibody-drug conjugates for cancer. Lancet.

[45] Khongorzul P., Ling C.J., Khan F.U., Ihsan A.U., Zhang J. (2020). Antibody-Drug Conjugates: A Comprehensive Review. Mol. Cancer Res..

[46] Hafeez U., Parakh S., Gan H.K., Scott A.M. (2020). Antibody-Drug Conjugates for Cancer Therapy. Molecules.

[47] Kline T., Steiner A.R., Penta K., Sato A.K., Hallam T.J., Yin G. (2015). Methods to Make Homogenous Antibody Drug Conjugates. Pharm. Res..

[48] Strop P., Liu S.H., Dorywalska M., Delaria K., Dushin R.G., Tran T.T., Ho W.H., Farias S., Casas M.G., Abdiche Y. (2013). Location matters: Site of conjugation modulates stability and pharmacokinetics of antibody drug conjugates. Chem. Biol..

[49] Holder P.G., Jones L.C., Drake P.M., Barfield R.M., Banas S., de Hart G.W., Baker J., Rabuka D. (2015). Reconstitution of Formylglycine-generating Enzyme with Copper(II) for Aldehyde Tag Conversion. J. Biol. Chem..

[50] Pan L., Zhao W., Lai J., Ding D., Zhang Q., Yang X., Huang M., Jin S., Xu Y., Zeng S. (2017). Sortase A-Generated Highly Potent Anti-CD20-MMAE Conjugates for Efficient Elimination of B-Lineage Lymphomas. Small.

[51] Hamblett K.J., Senter P.D., Chace D.F., Sun M.M., Lenox J., Cerveny C.G., Kissler K.M., Bernhardt S.X., Kopcha A.K., Zabinski R.F. (2004). Effects of drug loading on the antitumor activity of a monoclonal antibody drug conjugate. Clin. Cancer Res..

