# Supplementary figures and images for: EpCAM- and EGFR-Specific Antibody Drug Conjugates for Triple-Negative Breast Cancer Treatment
Source: Int J Mol Sci. 2022 May 30;23(11):6122. doi: 10.3390/ijms23116122 (PMC9181111; doi:10.3390/ijms23116122)

Figure S1

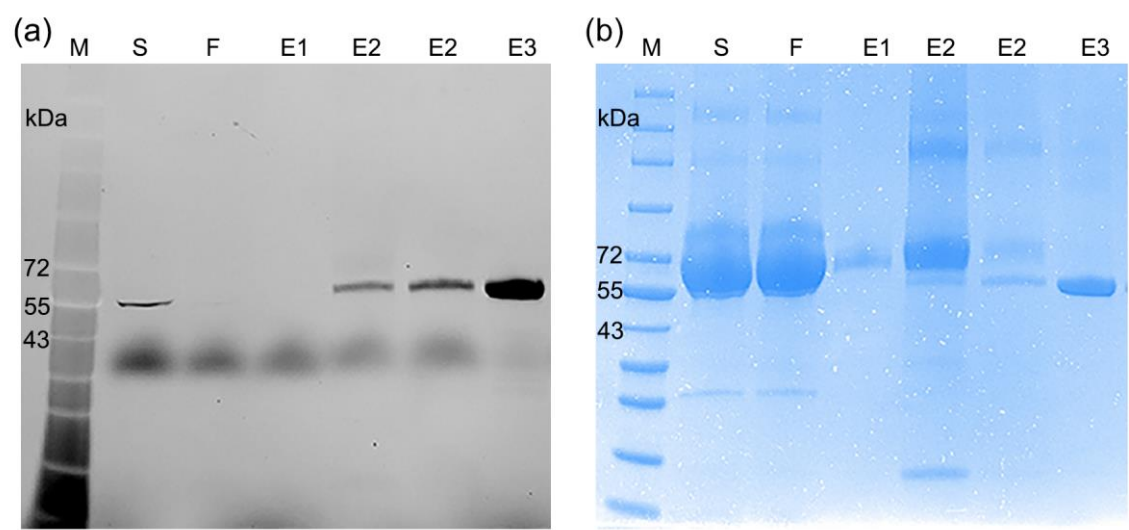

Figure S2

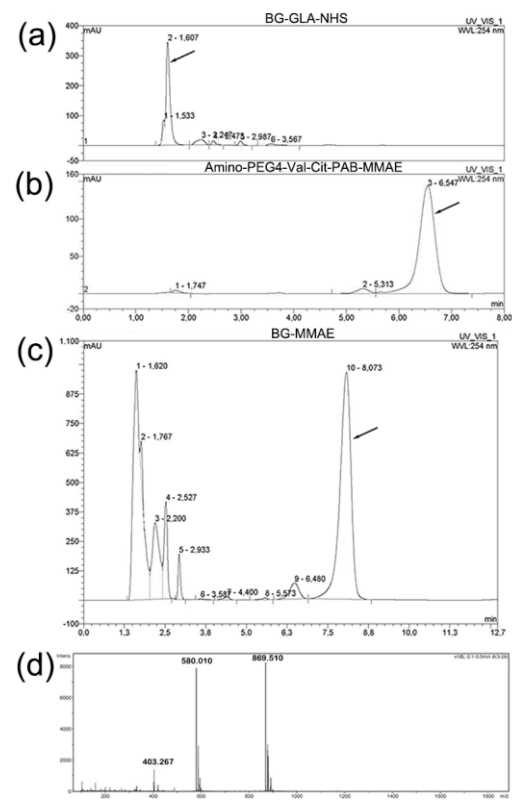

Supplement: Supplementary file 1 [file ijms-23-06122-s001.zip › ijms-1722250-supplementary.pdf]
